# Chemical Indicators of Self-Heating and Spontaneous Combustion in Stored Grains Investigated by HS-GC-MS

**DOI:** 10.1021/acsomega.6c03108

**Published:** 2026-08-07

**Authors:** Paula Baptista Leme, Gustavo Cabral Ferraz, Teresa Cristina Brazil Paiva, Maurício Lamano Ferreira, Eliane Corrêa Pedrozo, Flávio Teixeira da Silva

**Affiliations:** School of Engineering of Lorena, 119119 University of São Paulo (USP), Lorena, São Paulo 12602-810, Brazil

## Abstract

The self-heating and spontaneous combustion of grains represent a significant fire risk in agricultural storage systems, frequently occurring in the absence of external ignition sources. In this study, volatile organic compounds (VOCs) were investigated as chemical markers to reconstruct the evolution of the deterioration and thermal escalation processes associated with self-heating in stored corn. Gas chromatography with *headspace* sampling coupled to mass spectrometry (HS-GC-MS) was used to characterize the VOCs in samples from real fire scenarios and from simulation experiments under controlled conditions. The results suggested a progressive transition from biological deterioration to thermochemical degradation. The mapping of these chemical markers provided supportive evidence for the establishment of a timeline of thermal evolution, culminating in the signatures associated with *smoldering* combustion. These results highlight the potential of HS-GC-MS as a valuable analytical tool for the chemical reconstruction of fires involving stored grains, providing evidence that assists in the identification of self-heating processes and strengthens the scientific determination of the cause in agricultural storage incidents.

## Graphical abstract

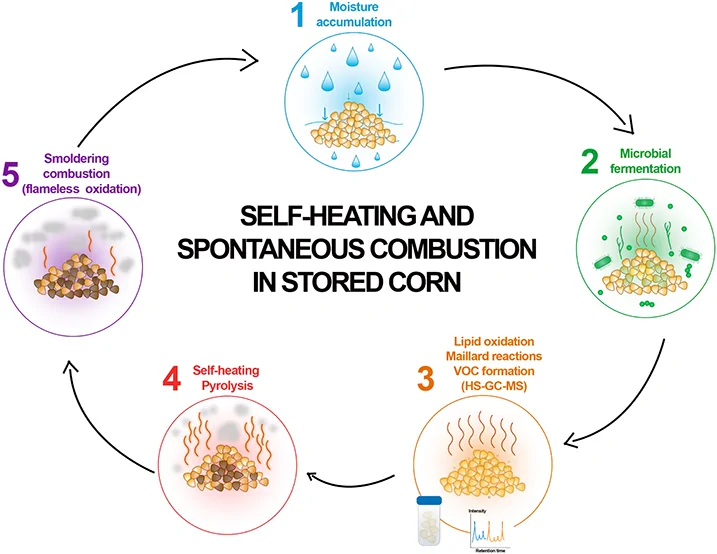

## Introduction

Fires in agricultural grain storage silos generally develop slowly and progressively, but pose significant risks, particularly due to the release of carbon monoxide (CO), a highly toxic and flammable gas. In addition to direct health risks, these fires are notoriously difficult for firefighting teams to control, owing to the large mass of combustible material, the structural design of the silos, and restricted access to the ignition zone.^1–5^

According to a survey conducted by Ferreira and Martinazzo,^5^ covering the period from 2011 to 2021, approximately 11% of accidents recorded in grain storage silos in Brazil were associated with fires or explosions. The highest concentration of these events occurred in the states of Mato Grosso and Rio Grande do Sul. The same study reported a significant increase of approximately 300% in the number of injured workers over the analyzed period, evidencing the growing severity and relevance of these incidents.

In this context, understanding the causes of fires and explosions in storage silos plays a crucial role in both the mitigation of operational risks and postincident investigation. In scenarios involving insurance coverage, technical investigations also provide essential support to insurers and claims adjusters, assisting in the classification of the event and the determination of coverage according to policy terms.

Among the mechanisms associated with fires and explosions in grain storage systems, the self-heating of the stored material stands out as one of the main triggers, frequently related to fermentative processes and the formation of potentially explosive atmospheres. High moisture contents, inadequate aeration, and microbial activity favor the generation of flammable gases, especially carbon monoxide, characteristic of *smoldering* combustion, a slow and flameless form of combustion.^1,2,6–14^

Spontaneous combustion is defined by NFPA 921 ^1^ as the initiation of combustion in a material through an internal chemical or biological reaction capable of generating sufficient heat to ignite the material itself. This definition applies directly to stored grains, such as corn, when conditions allow the internal accumulation of heat within the stored mass.

The self-heating process generally begins with the accumulation of moisture above the levels recommended for safe storage. Such conditions can result from inadequate practices, including a grain moisture content above 15%, insufficient air distribution inside the silo due to inadequate aeration, or exposure to environments with a relative humidity above 70%.^10,11^ These conditions create an ideal environment for microbial growth.

Subsequently, the presence of water and nutrients promotes microbial proliferation and triggers exothermic fermentative processes capable of raising the temperature of the stored material to values near 90 °C.^12,15–17^ The main types of fermentation observed under these conditions include alcoholic, acetic, and lactic fermentation, all contributing to heat generation and the chemical degradation of the grains.^12,15–17^

As the temperature continues to rise, nonenzymatic browning reactions, known as Maillard reactions, occur between amino acids or proteins and reducing sugars present in the grains, resulting in the formation of compounds such as hydroxymethylfurfural (HMF). These reactions are highly exothermic and are accelerated under conditions of low pH and elevated temperatures, typical of the preceding stages.^9,18^ In addition to contributing to thermal escalation, these reactions increase the content of fiber-bound proteins, such as acid detergent fiber (ADF) protein and acid detergent insoluble protein (ADIP), considered markers of advanced degradation. Experimental data indicate that, above 82 °C, ADIP levels increase exponentially, significantly intensifying the risk of ignition. In corn, the oxidation of oils present in the grains constitutes an additional source of exothermic heat.^19–27^

Furthermore, recent research indicates that low-temperature oxidation and the adsorption of water vapor (ambient moisture) significantly increase reaction rates, further promoting the thermal escalation that leads to spontaneous combustion.^28,29^

With the further increase in temperature and the progressive reduction of oxygen availability, pyrolysis begins, technically defined as the thermal degradation of organic compounds in the absence of oxygen, resulting in the formation of solid carbonaceous residues and the release of flammable gases.^13,14^ Due to the imperfect compaction of the stored grains, interstitial void spaces allow a continuous, albeit limited, permeation of oxygen into the core of the mass.^12,30^ This restricted oxygen supply is insufficient to sustain open flames but favors incomplete combustion, resulting predominantly in *smoldering*.^12^ This flameless process is characterized by the slow, heterogeneous oxidation of solid organic matter, continuous heat generation, greater release of carbon monoxide (CO), and the formation of carbonized, coal-like clusters.^30^

The risk of spontaneous combustion in agricultural storage silos is widely recognized in both scientific literature and regulatory standards. In Brazil, for example, the Fire Department of the State of São Paulo requires, through Technical Instruction No. 27/2018,^33^ the installation of thermometry systems in storage silos for the continuous monitoring of hot spots, with the objective of preventing autoignition events.

In fire investigations, physical and chemical evidence is fundamental for the development and validation of hypotheses related to spontaneous combustion.^1,2,6–8^ Typical indicators include the presence of carbonized clusters, elevated levels of carbon monoxide in the internal atmosphere of the silo, and anomalous records in thermal monitoring systems indicating localized heating.^1,2,4–14,30–32,34^

In recent years, the profiling of volatile organic compounds (VOCs) has emerged as a powerful strategy for monitoring grain quality and deterioration. While sensor arrays and electronic noses (E-noses) offer rapid screening, Gas Chromatography–Mass Spectrometry (GC-MS) remains the gold standard for the qualitative and structural elucidation of complex volatiles.^35,36^ Despite its high sensitivity, the analysis of spontaneous combustion processes by GC-MS presents limitations, particularly when applied to complex biomass matrices using spectral libraries instead of authentic analytical standards as references.^35^ Recognizing these limitations, this study employed HS-GC-MS as a comparative forensic tool by correlating the profiles of volatile organic compounds (VOCs) detected as physical evidence in real large-scale silo fires. This work established a novel forensic approach to chemically reconstruct the self-heating timeline, mapping the transition from biological deterioration to smoldering combustion.

## Experimental Section

### Case Studies and Sample Collection

This study analyzes physical evidence collected from two real fires in corn storage silos, in which spontaneous combustion was considered the probable cause of the event. Due to confidentiality requirements, specific details regarding the location and identification of the silos are not disclosed.

In addition to the samples collected from fire incidents, a reference sample (control) was obtained from a distinct processing unit. This sample consisted of intact, having been stored for only a few days after processing and drying, presenting a moisture content of 9.88%. As shown in Figure 1, the grain exhibited high quality without evidence of degradation. This standard was used as a reference to map the volatiles background, allowing for the exclusion of volatile organic compounds (VOCs) naturally emitted by healthy, recently processed corn.

**Figure 1:**
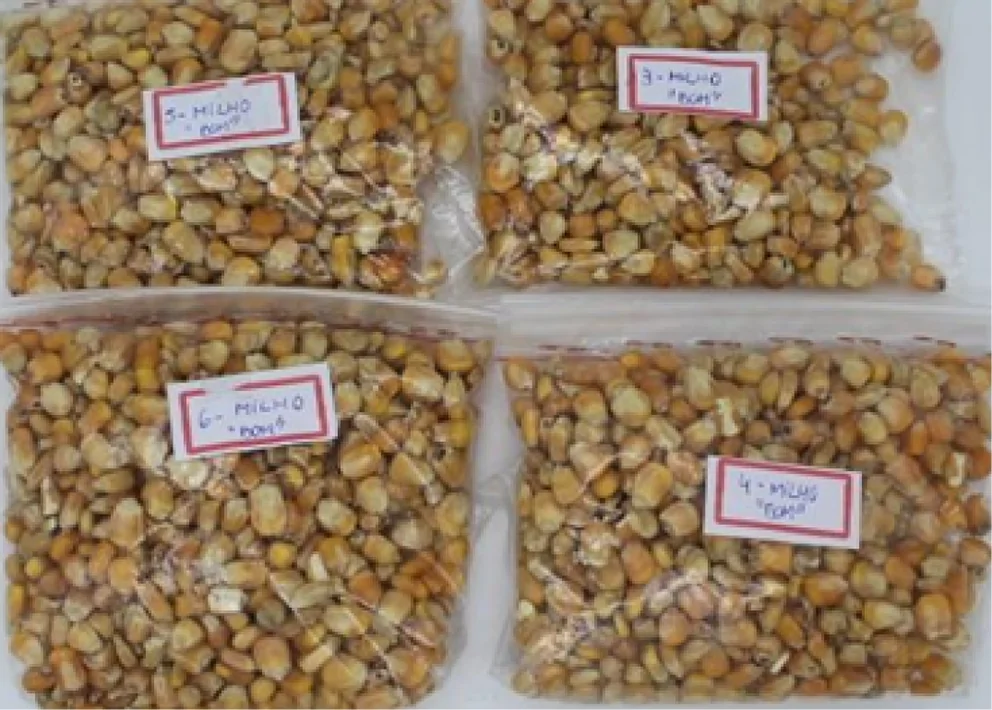
Representative image of the field reference (control) corn sample collected from a nonaffected silo shortly after processing and drying (moisture content: 9.88%), showing intact corn without visible signs of biological or thermal degradation.

In the first case study, representative samples of three distinct conditions of the stored material were collected: visually intact corn grains, sampled from a distant area within the same silo (conveyor belt), containing approximately 5% charred grains; partially carbonized corn grains (collected from the top of the silo), whose sample consisted predominantly of noncarbonized grains, with approximately 15% carbonized clusters, suggesting localized thermal exposure without complete biomass carbonization; and fully carbonized corn clusters (collected from the fire’s zone of origin). These samples presented clear thermal degradation gradients, ranging from grains with no apparent damage to consolidated carbonized masses, with typical characteristics of slow and flameless combustion (smoldering). Figure 2 presents the samples to Case Study 1.

**Figure 2:**
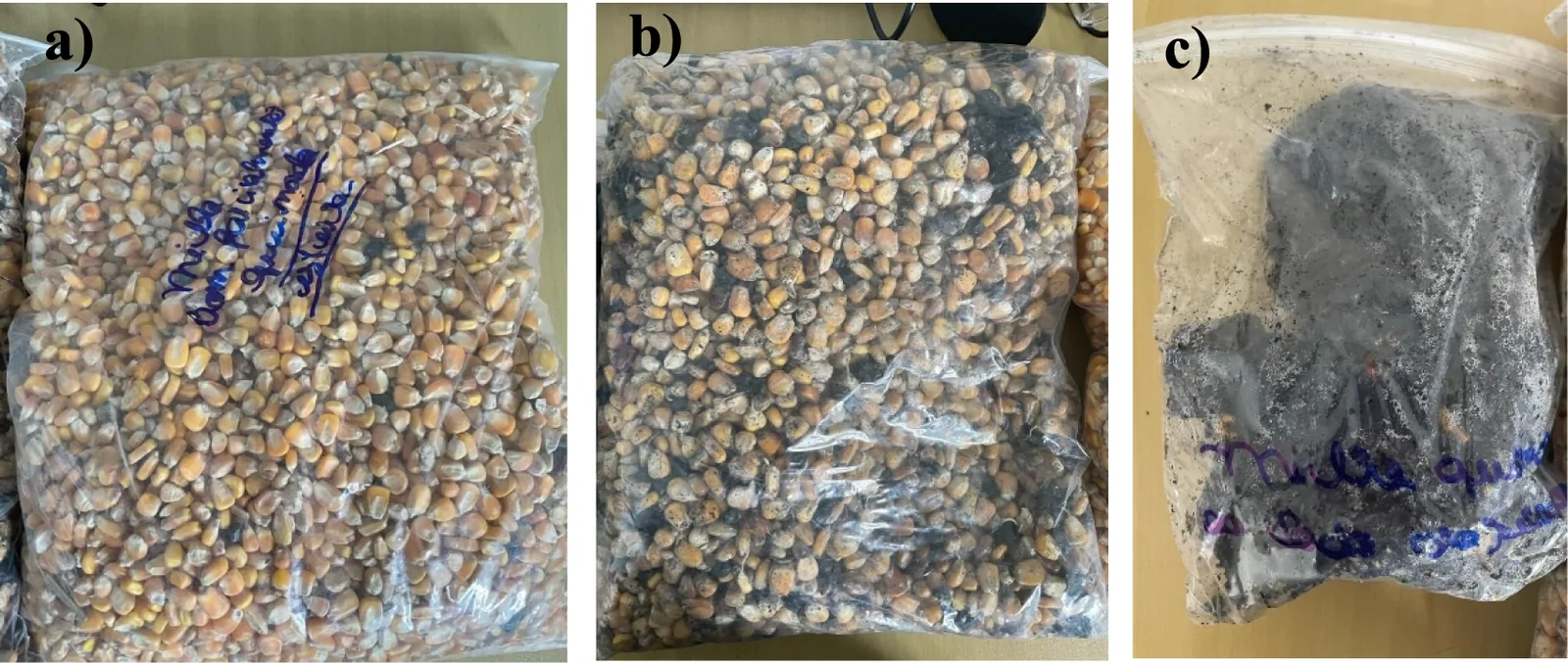
Samples collected in Case Study 1: (a) visually intact corn grains, sampled from a distant area within the same silo (conveyor belt), containing approximately 5% charred grains (b) partially burned sample, and (c) burned sample.

In the second case study, it was not possible to sample dry and intact corn from the silo. The collected corn presented a high moisture content (estimated at 40%, well above the 15% maximum limit recommended for safe storage), with visible signs of active fermentation (detected by visual and olfactory inspection, such as turbidity and water condensation on the package walls). The second sample consisted of partially burned corn grains (PB) with evidence of thermal degradation. Visual analysis of this PB sample revealed grayish coloration and evidence of advanced fermentation, without the presence of completely carbonized grains. This physical aspect indicated that the material had undergone intense thermal and oxidative stress without complete pyrolytic carbonization. The samples referring to Case Study 2 are presented in Figure 3.

**Figure 3:**
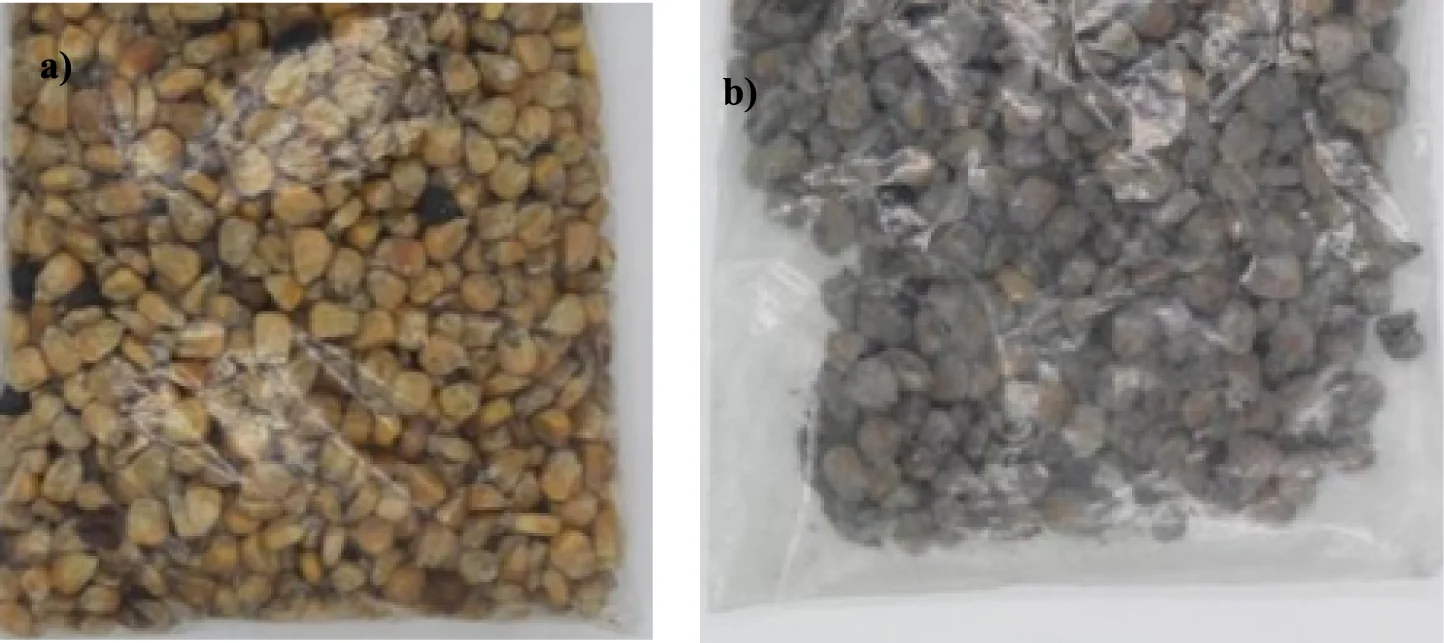
Samples collected in Case Study 2: (a) wet corn grains with visible signs of fermentation and (b) partially burned corn grains presenting thermal degradation.

### Fermentation Simulation

For case study 1, fermentation simulation experiments were conducted using corn samples visually intact corn grains isolated from the conveyor belt sample, with approximately 9% moisture. A mass of 10 g of milled corn was transferred to a 250 mL glass flask, followed by the addition of 10 mL of distilled water. The flask was sealed and maintained at 40 °C for 10 days to simulate fermentation under controlled conditions. This specific incubation period and temperature were selected because 40 °C corresponds to the optimal growth range for the inherent deteriorating microbiota, and the 10-day period provides sufficient time to ensure the transition from initial aerobic respiration to anaerobic fermentation, promoting the accumulation of primary volatile biomarkers (such as alcohols and short-chain organic acids) without artificially forcing advanced thermal degradation. All fermentation experiments were conducted in a New Lab NL 82-480 laboratory incubator.

It is important to note that the laboratory simulations presented herein are simplified models designed strictly to support the proposed chemical interpretations, rather than full scale reproductions of the complex and dynamic scenarios found in real silo fires.

Figure 4 presents the sample after the fermentation simulation, in which visible changes associated with the fermentative process were verified after the test, such as moisture condensation on the flask walls, change in grain coloration, and water turbidity.

**Figure 4:**
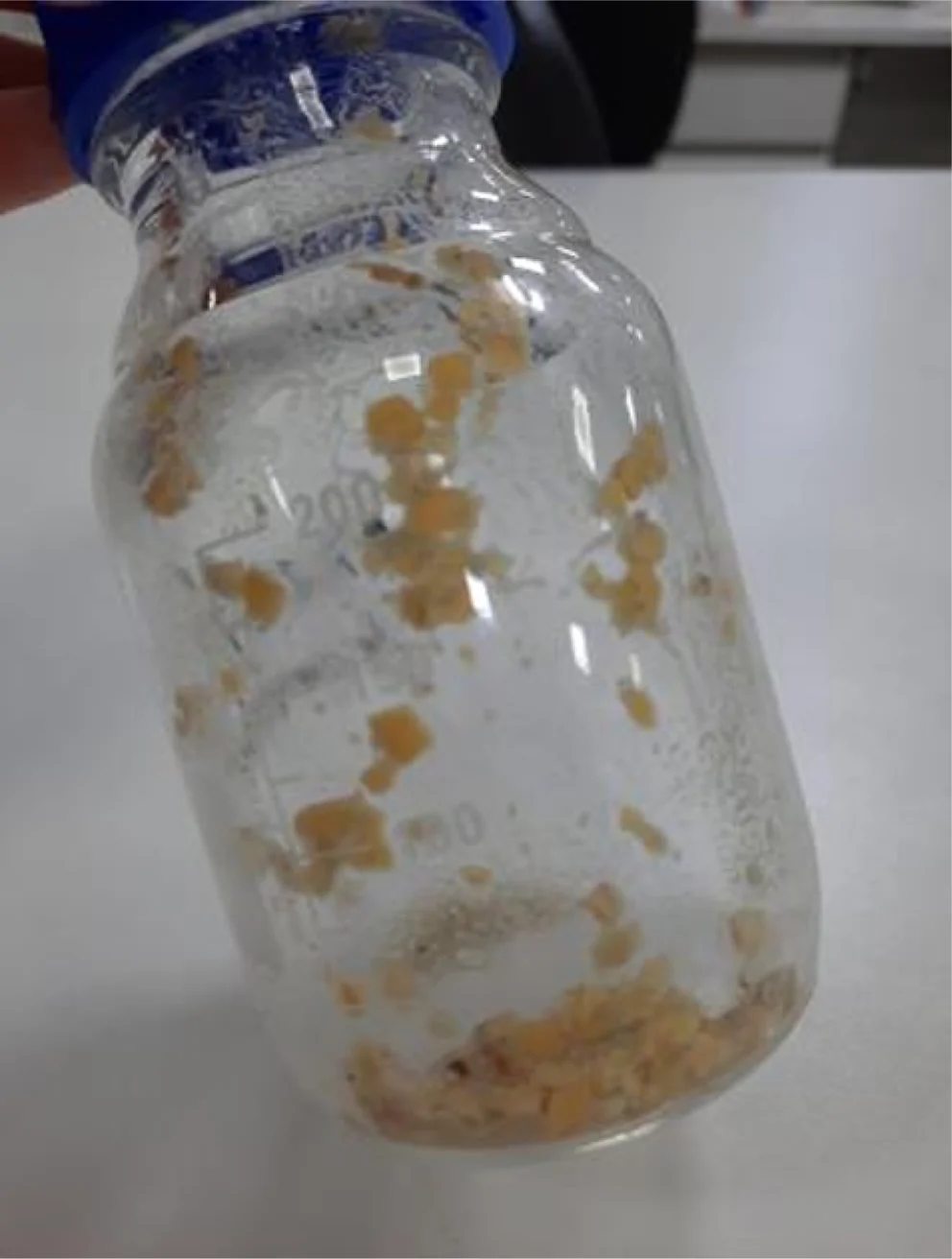
Corn sample after the fermentation simulation test conducted under controlled temperature conditions, presenting visible changes associated with the fermentative process.

### Thermal Stress Simulation

For Case Study 2, 13.5 g of corn with a high moisture content were subjected to a thermal stress simulation in an open Petri dish without the addition of water, for periods of 3 and 7 days, at a temperature of 60 °C. This temperature was selected because the incident occurred in a grain dryer silo that operated at approximately 60 °C.

The use of an open system allowed for the gradual evaporation of moisture, thereby inhibiting prolonged microbial fermentation and successfully simulating the subsequent phase of the spontaneous combustion timeline: thermal stress and lipid oxidation (visual comparisons of the samples during the accelerated aging process are provided in Figure S7 of the Supporting Information).

### Gas Chromatography with *Headspace* Sampling Coupled to Mass Spectrometry (HS-GC-MS)

Volatile organic compounds (VOCs) were analyzed by gas chromatography with *headspace* sampling coupled to mass spectrometry (HS-GC-MS). The *headspace* technique allows the analysis of volatile components released from the sample matrix after heating in a hermetically sealed vial, minimizing sample handling and preserving the original chemical profile.

The GC-MS analyses were performed using a Shimadzu QP-2010 gas chromatograph coupled to a TQ-8040 mass spectrometer. The mass spectrometer operated in full-scan mode, in the *m*/*z* 40–700 range. Helium was used as the carrier gas, with a split ratio of 1:5. All analyses were conducted under controlled instrumental conditions, appropriate for the analysis of volatile organic compounds by *headspace*.

Compound identification was performed by comparing the obtained mass spectra with reference data from the NIST 17 spectral library, applying a minimum spectral similarity threshold of 80%. Considering the inherent limitations of identification based exclusively on spectral library-matching, especially in complex biomass matrices, the compounds were classified as tentative identifications.^35,36^ This analytical approach provides a reliable assignment of chemical classes and is widely employed in exploration and comparative studies, particularly in forensic investigations based on volatile organic compound (VOC) profiling. However, unequivocal structural confirmation, including the exact distinction between isomers, requires the analysis of authentic analytical standards and/or the calculation of additional orthogonal parameters, such as retention indices (RI).^35^ Detailed instrumental parameters are presented in the Supporting Information.

For sample preparation, 2.0 g of each material were individually transferred into 20 mL *headspace* vials, sealed, and subjected to analysis. In Case Study 1, the following samples were analyzed: intact corn, corn after fermentation simulation, partially burned corn, and carbonized corn with *smoldering* characteristics. All analyses were performed in duplicate for the fermentation simulation (Figure 5).

**Figure 5:**
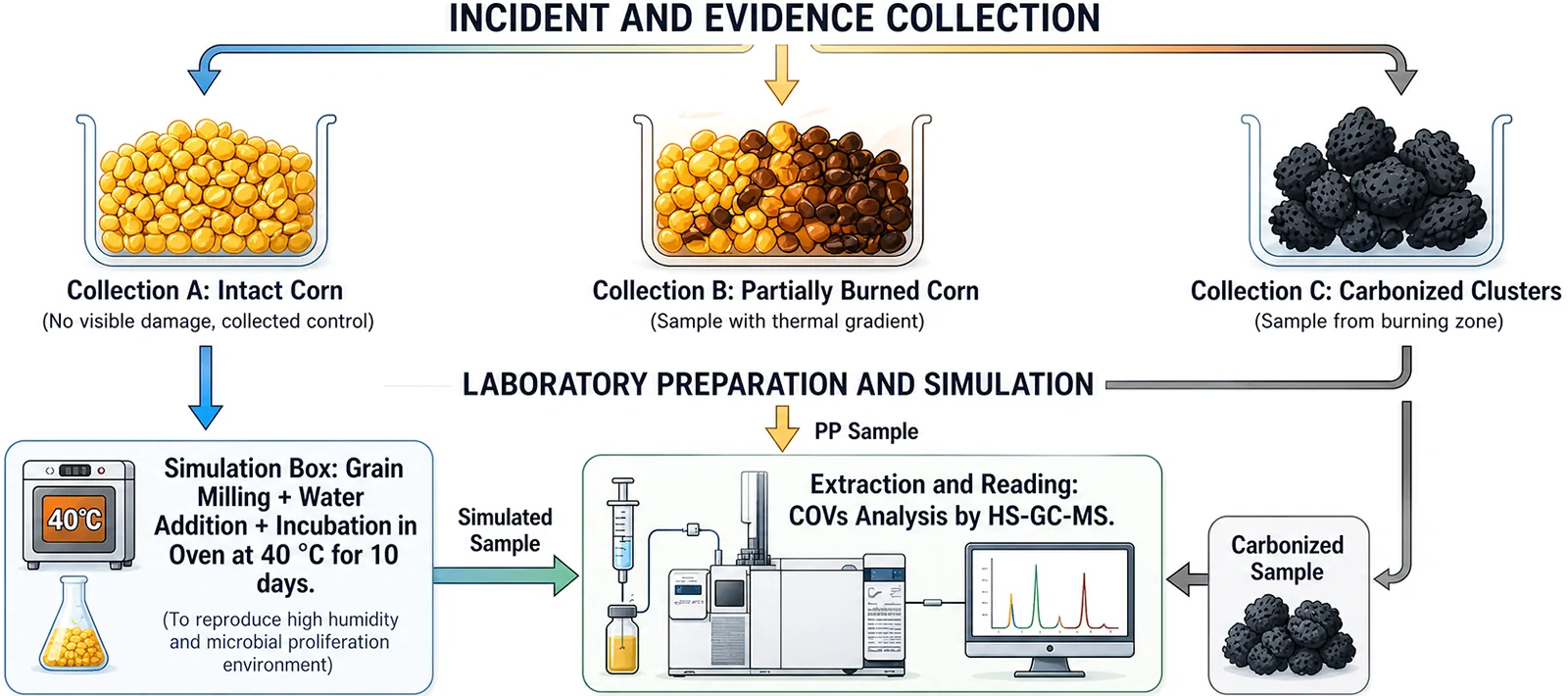
Representative forensic flowchart of the experimental design of Case Study 1. The scheme illustrates the evidence collection strategy with a thermal gradient, the accelerated laboratory simulation step at 40 °C, and the analytical protocol for VOC extraction by HS-GC-MS.

For Case Study 2, the HS-GC-MS analyses were performed in a single injection, and the results represent the relative abundance (in area percentage) of the obtained chromatographic peaks (Figure 6).

**Figure 6:**
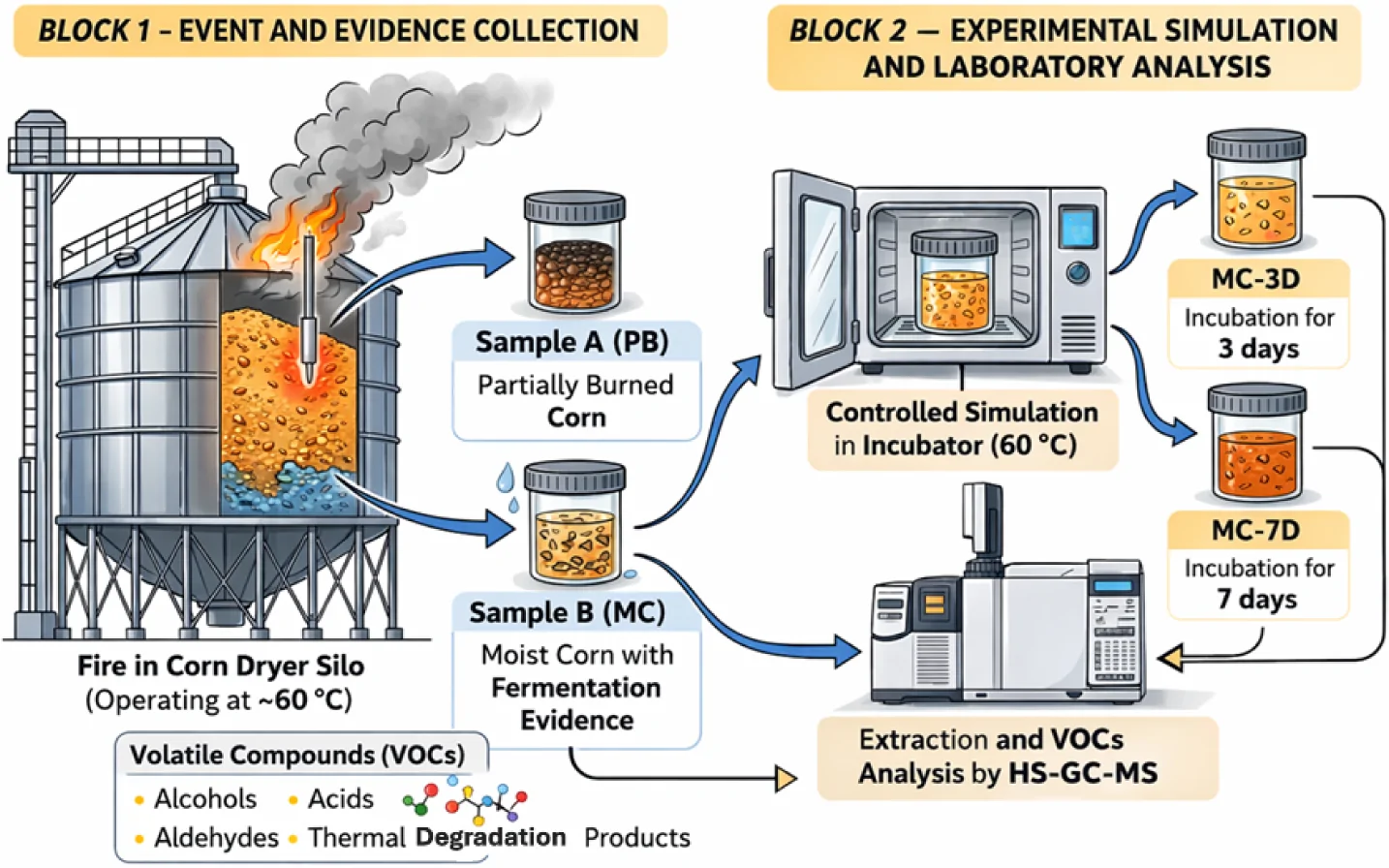
Study 2: the illustration details the event in the dryer silo, the collection of samples subjected to the accelerated aging procedure in an oven at 60 °C, followed by HS-GC-MS analysis.

## Results and Discussion

### Identification of Biomarkers in the Control Sample

The HS-GC-MS profile of the field reference (intact corn collected from a nonaffected silo shortly after processing and drying, with 9.88% of humidity) was evaluated. As illustrated by the detailed chromatograms provided in Figure S3 of the Supporting Information, the profile of this reference corn exhibited a behavior identical to the analytical blank. The few peaks detected in both chromatograms corresponded exclusively to siloxanes, which are well-known instrumental artifacts derived from the degradation of the stationary phase of the chromatographic column (column bleed). The complete absence of other signals confirms that this baseline sample lacked the characteristic short-chain aldehydes, alcohols, organic acids, and nitrogenous compounds detected in the deteriorated samples.

### Case Study 1: VOCs Associated with Fermentation and Thermal Degradation

Table 1 summarizes the volatile organic compounds (VOCs) identified by gas chromatography with *headspace* sampling coupled to mass spectrometry (HS-GC-MS) in the samples collected in Case Study 1. The analyzed materials included corn subjected to a 10-day fermentation simulation, partially burned corn collected at the fire scene, and carbonized clusters associated with *smoldering* combustion. The fermentation simulation analyses were performed in duplicate, and the presented values correspond to the obtained means standard deviations.

**Table 1:** HS-GC-MS Results for Case Study 1 Corn Samples after Fermentation Simulation, Partially Burned Corn, and Carbonized Clusters^a^

Compound	Corn after fermentation simulation (area %)	Partially burned corn (area %)	Carbonized clusters (area %)
3-Methylbutanal	4.1 ± 0.2	22	ND
2,4,5-Trimethyl-1,3-dioxolane	28 ± 2	1.5	ND
3-Methyl-1-butanol	68 ± 2	42	ND
2-Methylbutanal	ND	4.6	ND
Ethyl propanoate	ND	8.9	ND
Isobutyl acetate	ND	4.7	ND
2-Propenyl butanoate	ND	2.6	ND
Lactic acid (tentatively identified)	ND	6.8	ND
Pentyl acetate	ND	6.4	ND

a ND = not detected under the applied analytical conditions. The relative mass percentages were calculated based on the peak areas obtained by HS-GC-MS.

The samples subjected to the fermentation simulation presented VOCs predominantly belonging to the aldehyde, alcohol, and cyclic acetal classes. The presence of alcohols (such as 3-methyl-1-butanol) in these samples is consistent with microbial amino acid degradation processes (Ehrlich pathway) and early fungal activity, which act as primary indicators of biological spoilage.^17,31^ Consistently, the detection of similar compounds in the partially burned corn, associated with the additional presence of organic acids (such as lactic acid) and esters, suggests that fermentation was already occurring inside the silo prior to the fire’s ignition. The presence of both compounds (alcohol and lactic acid) also indicates that the progressive reduction of oxygen within the densely packed grain mass may have favored the evolution of the process to a predominantly anaerobic deterioration stage, characterized by lactic fermentation and the formation of lactic acid.^3,17^

Although lactic acid generally exhibits relatively low volatility and a melting point that varies depending on its chemical form, its tentative detection was feasible under the applied analytical conditions.^35^ In the HS-GC-MS technique, heating the sample in a hermetically sealed vial promotes the partitioning of volatile compounds into the gas phase. Upon injection, the sample is completely vaporized in the heated injector (e.g., 200–300 °C) and continuously transported by the carrier gas. This enables the compound to travel through the chromatographic column and be properly detected, despite the initial GC oven temperature being set at 40 °C, which is below the melting point of its pure solid form. Furthermore, lactic acid may be present as a mixture of its l- and d-enantiomers (racemic mixture), a condition that significantly lowers its melting point compared to the pure enantiomers, potentially to approximately 16–18 °C.^36^ Therefore, the presence of lactic acid in the liquid phase or its volatilization under the analytical conditions employed becomes even more plausible.

The detection of 2,4,5-trimethyl-1,3-dioxolane in the fermented samples provided crucial insights into the chemical dynamics within the silo. Rather than being a direct microbial metabolite, this cyclic acetal is typically formed through acid-catalyzed condensation (acetalization) between acetaldehyde and 2,3-butanediol, a well-documented metabolite naturally produced by *Saccharomyces cerevisiae* and other yeasts during grain fermentation.^37^ Although the presence of this acetal in the literature remains limited,^46^ recent studies on spontaneously fermented maize-based matrices, such as the traditional beverage Munkoyo, have confirmed its occurrence.^38^

Therefore, it is inferred that the heated and acidic environment within the deteriorating grain mass acts as a reactive medium, favoring secondary nonenzymatic transformations from primary microbial precursors. This proposition is supported by the presence of 2,4,5-trimethyl-1,3-dioxolane in significant amounts in the corn samples subjected to fermentation (28%) (Table 1). In contrast, in the partially burned sample, this compound was detected at substantially lower levels (1.5%), which may be interpreted as a residual product of the fermentation process described above. It is noteworthy that, in the control sample, the presence of this compound, as well as the other compounds described in Table 1, was absent or below the detection limit of the chromatographic method employed.

The complete absence of lactic acid and secondary esters (such as ethyl propanoate and pentyl acetate) in the laboratory simulation after 10 days confirmed that these compounds are not primary fermentation products (Table 1). Their formation requires severe oxygen depletion, prolonged reaction time, and the accumulation of thermal energy, characteristics of advanced deterioration stages within the massive structure of a real silo.^3,31^ In this highly confined environment, the initial heat generated by microbial metabolism cannot dissipate, acting as a thermal trigger that accelerates secondary esterification reactions.^8,12^

Furthermore, the variations observed in these shared markers strongly corroborate the advanced stage of self-heating in the real silo. For example, 3-methylbutanal exhibited an increase greater than 400% in the partially burned sample (22%) compared with the simulated fermentation sample (4%) (Table 1). Although initially produced through microbial metabolism, its pronounced accumulation in the fire sample is driven by the heat-induced Strecker degradation of amino acids during advanced Maillard reactions.^39,40^ In contrast, primary biological markers, such as 3-methyl-1-butanol and 2,4,5-trimethyl-1,3-dioxolane, were detected in lower concentrations in the partially burned sample. This reduction may indicate that, as the thermal escalation progressed, microbial biosynthesis ceased and the primary alcohols were either volatilized or consumed in secondary esterification reactions.^8,12,31^

In contrast, no volatile compounds were detected in the carbonized samples by HS-GC-MS, indicating extensive volatilization resulting from exposure to high temperatures during *smoldering* combustion.

### Case Study 2: Chemical Markers from Oxidation and *Smoldering*

It should be emphasized that the data from Case Study 1 correspond to area percentages obtained from duplicate analyses only for the fermentation simulation, whereas the data from Case Study 2 correspond to area percentages derived from a single injection. Therefore, the comparative analysis between the two case studies presented herein is strictly qualitative. Accordingly, the discussion focused exclusively on the presence, absence, and evolution of the chemical classes, including biological and thermal markers, rather than on direct absolute quantitative comparisons.

The HS-GC-MS results obtained for Case Study 2 are presented in Table 2 and include the analysis of: (i) partially burned corn collected at the fire scene, (ii) corn with high moisture content, (iii) corn with high moisture content after fermentation simulation at 60 °C for 3 days, and (iv) corn with high moisture content after fermentation simulation at 60 °C for 7 days.

**Table 2:** HS-GC-MS Results for Case Study 2 Corn Samples, Including Partially Burned Corn (PB); Wet Corn Sampled from the Silo (MC), Wet Corn after 3 Days of Accelerated Aging (MC-3D); Wet Corn after 7 Days of Accelerated Aging (MC-7D)

Compound	PB (area %)	MC (area %)	MC-3D (area %)	MC-7D (area %)
Neopentyl acetate	ND	5.08	ND	ND
Hexanal	ND	4.54	ND	28.54
2,3-Dimethyl-2-cyclopentenone	5.10	2.59	ND	ND
2-Methylphenol	2.49	2.37	ND	ND
4-Ethyl-2-methoxyphenol	2.53	ND	ND	ND
2,3,6-Trimethylpyridine	7.19	ND	ND	ND
2-Ethyl-4,6-dimethylpyridine	3.53	ND	ND	ND
Tetramethylpyrazine	11.15	ND	ND	ND
2,5-Dimethyl-6,7-dihydro-(5H)-cyclopentapyrazine	2.51	ND	ND	ND
11,14-Eicosadienoic acid	1.50	ND	ND	ND
Hexadecanenitrile	1.96	ND	ND	ND
Unidentified compounds	13.92	49.60	39.69	35.16
Nonanal	ND	5.33	12.17	17.95
Naphthalene	ND	2.98	15.73	5.34
1-Methylnaphthalene	ND	1.37	ND	ND
1-Dodecanol	ND	2.22	ND	ND
Ethyl hexadecanoate	ND	1.55	ND	ND
Ethyl linoleate	1.26	2.68	4.96	ND
Ethyl oleate	1.16	2.59	2.77	ND

The partially burned corn sample presented a significant diversity of volatile organic compounds. Among the identified compounds, substituted phenols stand out, such as 2-methylphenol and 4-ethyl-2-methoxyphenol (homoguaiacol), frequently associated with the thermal degradation of phenolic compounds and phenolic acids present in the plant cell wall. The presence of these compounds results from partial pyrolysis processes of the plant biomass during heating.^41^

Nitrogenous heterocyclic compounds were also detected, including 2,3,6-trimethylpyridine, 2-ethyl-4,6-dimethylpyridine, tetramethylpyrazine, and 2,5-dimethyl-6,7-dihydro-(5H)-cyclopentapyrazine. The formation of these compounds is associated exclusively with advanced thermochemical processes, particularly high-temperature Maillard reactions and the Strecker degradation of amino acids and reducing sugars during the severe heating of the organic material.^33,34,46^

Furthermore, compounds such as 11,14-eicosadienoic acid, a polyunsaturated fatty acid, and hexadecanenitrile, a nitrile derived from palmitic acid, were identified. While these may result from the thermal degradation of lipids present in the grain.^35,41^

The corn samples with high moisture content and those subjected to accelerated aging at 60 °C (MC-3D and MC-7D) presented a greater number of peaks with higher intensities associated with unidentified volatile compounds when compared to the partially burned sample (PB). In the MC sample, VOCs associated with fermentative processes were identified. In contrast, these compounds were not detected in the MC-3D and MC-7D samples, indicating the absence of microbial activity. During the accelerated aging process, ethyl oleate and ethyl linoleate were detected in all samples except MC-7D. The absence of these esters may be explained by the auto-oxidation of linoleic acid, accompanied by the formation of hexanal (28%) and nonanal (17.95%), which are well-established biomarkers.^18,39,40^

In contrast, the partially burned corn (PB) exhibited a completely distinct and exclusive chemical signature, dominated by nitrogenous heterocyclic compounds (e.g., tetramethylpyrazine, 2,3,6-trimethylpyridine, and 2-ethyl-4,6-dimethylpyridine), substituted phenols (2-methylphenol and 4-ethyl-2-methoxyphenol), and advanced thermal markers (2,3-dimethyl-2-cyclopentenone and hexadecanenitrile). The absence of these compounds in the experiments conducted at 60 °C suggests that they are not primarily generated solely by moderate thermal stress. In this context, substituted phenols likely indicate the pyrolytic cleavage of organic matter,^1,4^ whereas complex pyrazines and pyridines are generally associated with advanced Maillard reactions and Strecker degradation at higher temperatures.^33,34,46^

On the other hand, the detection of naphthalene and 1-methylnaphthalene in the moist and aged samples (MC, MC-3D, and MC-7D) may be interpreted as resulting from the adsorption of compounds formed during fire-induced pyrolysis. The layers of unburned grains with high moisture content located at the upper region of the silo likely acted as a filter.

Furthermore, this thermochemical behavior fully explains the absence of hexanal, complex esters, and furans in the control sample. In intact grains, the lipid fraction is physically protected within the cellular matrix. However, as the initial biological deterioration and subsequent thermal degradation progress, the cellular microstructure undergoes collapse, exposing the lipids.^31^ This structural breakdown constitutes the prerequisite that accelerates the homolytic cleavage and auto-oxidation of polyunsaturated fatty acids.^18^ Therefore, the notable relative presence of hexanal and oxidative markers does not merely represent a chemical finding, but an indicative marker of the extensive physical degradation of the grain matrix, which inevitably precedes thermal escalation and ignition.^8,12^

Finally, the partially burned corn collected from the incident revealed the most advanced stage of this degradation, dominated by severe pyrolysis markers such as furans and substituted phenols.^41^ This profound chemical shift corroborates the self-heating timeline progressing from localized heating to active smoldering combustion.^8,12^

### Chemical Interpretation and Implications for Self-Heating

The comparative analysis of the VOC profiles obtained by HS-GC-MS reveals distinct chemical signatures that allow the chronological mapping of the biological, physical, chemical, and thermochemical phases of the self-heating process. The predominance of markers of biological origin in the Case Study 1 samples reflects the analytical conditions employed, characterized by high water activity and a moderate temperature of 40 °C, which simulated the initial stages of moisture accumulation in a silo. In this environment, metabolic pathways associated with amino acid degradation and alcoholic fermentation were favored, justifying the presence of compounds such as 3-methyl-1-butanol. As oxygen was progressively depleted within the real silo, this initial biological phase evolved into an intense anaerobic deterioration stage prior to ignition.^29^ This transition is chemically evidenced by the exclusive accumulation of lactic acid and the subsequent formation of secondary volatile esters, such as ethyl propanoate and pentyl acetate.^17^

In turn, the accelerated aging simulation (60 °C) of Case Study 2 adequately models the transition the transition from predominantly metabolic processes to heating-induced chemical reactions.^28^ As the thermal stress progresses and microbial activity is inhibited, the lipids naturally present in the corn undergo accelerated oxidative degradation. The temporal evolution demonstrates that lipid esters are gradually cleaved into short-chain aldehydes, especially hexanal and nonanal, which act as primary markers of this thermal escalation.^18^

As self-heating reaches critical temperatures, more advanced thermochemical degradation of the biomass matrix occurs.^13,28^ Marked differences in the VOCs composition between the laboratory simulations and the real partially burned samples provide a chemical timeline of the incident. In these advanced stages, the detection of nitrogenous heterocyclic compounds (such as pyridines and tetramethylpyrazine) confirms their predominantly thermal origin, driven by extreme Maillard reactions and Strecker degradation at high temperatures.^39,44,46^

Concurrently, the formation of substituted phenols, such as 2-methylphenol and 4-ethyl-2-methoxyphenol (homoguaiacol), derives from the partial pyrolysis of phenolic compounds present in the plant cell wall.^41^

In contrast, the absence of volatile compounds in the fully carbonized clusters observed in Case Study 1 evidence the intense thermal volatilization of the organic matrix subjected to the high temperatures of the active combustion zone.^12,13^

A further detailed analysis of the thermochemical pathways synthesized in Tables 3 and 4 elucidates the apparent absence of light alcohols (e.g., ethanol and propanol) during the early stages of fermentation, an aspect that warrants careful forensic consideration. While short-chain alcohols are typical primary metabolites of yeast, their nondetection in the advanced sampling stages is scientifically justified by their volatility and the system’s thermodynamics. In the open-system simulations and during the delayed field collection of the heated silo samples, these highly volatile compounds rapidly dissipate into the surrounding atmosphere. Furthermore, under aerobic or semiaerobic conditions, transient light alcohols are rapidly consumed by acetobacteria and oxidized into acetic acid, a robust secondary marker that was prominently detected in our analyses.^17^

**Table 3:** Correlation of the Compounds Detected by HS-GC-MS with the Spontaneous Combustion Stages in Case Study 1^a^,^b^,^c^

Process stage	Detected chemical indicators	Evidence in samples	Chemical interpretation and mechanism	References
**Alcoholic fermentation and amino acid degradation**	3-Methyl-1-butanol; 3-Methylbutanal;2,4,5-Trimethyl-1,3-dioxolane	Simulation and PB	3-Methyl-1-butanol and 3-methylbutanal derive from amino acid conversion (Ehrlich pathway). 2,4,5-Trimethyl-1,3-dioxolane is a secondary formation driven by the acid-catalyzed condensation of microbial 2,3-butanediol with acetaldehyde during advanced anaerobic deterioration. Furthermore, highly volatile short-chain alcohols (e.g., ethanol) produced during this stage likely dissipated into the headspace during the prolonged 10-day incubation or were rapidly consumed.	^37–39,42,43^
**Lactic fermentation and advanced anaerobic deterioration**	Lactic acid (tentatively identified); 2-Methylbutanal	PB (Only at the fire scene)	The presence of lactic acid indicates a transition to intense anaerobic microbial activity, oxygen depletion, and a more advanced deterioration stage than the one simulated in the laboratory.	^39^
**Initial esterification reactions**	Ethyl propanoate; Isobutyl acetate; Pentyl acetate; 2-Propenyl butanoate	PB (Only at the fire scene)	Complex secondary formation resulting from the reaction between fermentation alcohols and organic acids released with the initial temperature increase.	^39,42^
			The significant detection of these esters provides indirect proof of early short-chain alcohol generation.	
***Smoldering*** **combustion and carbonization**	Absence of volatile organic compounds (ND)	Carbonized clusters	Extensive volatilization and total thermochemical breakdown of VOCs resulting from sustained exposure to the high temperatures of *smoldering*.	^28,39^

a PB = partially burned corn.

b MC-3D = wet corn after 3 days of accelerated aging (thermal stress).

c MC-7D = wet corn after 7 days of accelerated aging (thermal stress).

**Table 4:** Correlation of the Compounds Detected by HS-GC-MS with the Spontaneous Combustion Stages in Case Study 2

Process stage/Class	Detected chemical indicators	Evidence in samples	Chemical interpretation and mechanism	References
Abiotic Thermal Trigger (Lipid Auto-oxidation)	Hexanal; Nonanal	MC-3D, MC-7D	Primary products of the homolytic cleavage of lipid hydroperoxides induced by the initial abiotic thermal stress (60 °C).	^18,39^
Thermochemical cell wall degradation (Lignin Pyrolysis)	2-Methylphenol; 4-Ethyl-2-methoxyphenol	PB	Pyrolytic cleavage of lignin and phenolic polymers from the plant cell wall at extreme temperatures.	^41^
Advanced Maillard reactions and Smoldering	Tetramethylpyrazine; 2,3,6-trimethylpyridine; 2-ethyl-4,6-dimethylpyridine; 2,5-Dimethyl-6,7-dihydro-(5H)-cyclopentapyrazine	PB	Predominantly thermal origin. Formed by extreme Maillard cascade reactions and Strecker degradation of amino acids at high temperatures, characteristic of the transition from thermal runaway to active smoldering.	^39,44,46^
Advanced thermochemical degradation	Hexadecanenitrile	All samples/PB	Cleavage products of lipid hydroperoxides and advanced oxidative/thermal degradation (pyrolysis) of organic matter.	^28,39,41^

Finally, the HS-GC-MS acquisition parameters are fundamentally optimized to detect heavier, more persistent biomarkers (such as 1-pentanol and 1-hexanol) that remain entrapped within the biomass matrix. Therefore, the absence of light alcohols does not contradict the biological trigger hypothesis; rather, it highlights the continuous biochemical conversion and volatilization processes that characterize the transition to the thermal runaway phase.

Despite the proposed timeline, the interpretation of these VOCs requires caution. Establishing specific and universal compounds as definitive markers of spontaneous combustion remains a challenge.^28^ Thermochemical and biological processes in complex matrices, such as corn, involve overlapping mechanisms.^28,35^ For example, environmental variations, localized oxygen availability, and the microbiome inherent to the environment in which the sample is found may cause significant changes in the volatile compounds.^29,40^ Therefore, rather than treating individual VOCs as definitive and isolated evidence, the chemical signatures discussed in this study should be interpreted as part of a broader forensic tool supporting the self-heating hypothesis.^1,6,8^ Future quantitative studies using authentic analytical standards and multidimensional chromatography are recommended to further reduce these analytical uncertainties.^35,36^

Together, this chemical mapping, from moisture accumulation and microbial fermentation to lipid oxidation and biomass pyrolysis, allows the reconstruction of the chemical timeline of the event. The proposed biochemical reaction mechanism is synthesized in Table 3 and illustrated in Figure 7 for Case Study 1, as well as in Table 4 and Figure 8 for Case Study 2. These chemical markers provide a scientific basis for interpreting the origin of the fire through the intrinsic self-heating of the biomass. However, recognizing the inherent uncertainties of complex fire scenes, the definitive exclusion of external ignition sources strictly depends on the broader investigative context and corroborating documentary evidence. Therefore, these findings demonstrate the applicability of HS-GC-MS not as a standalone proof, but as a powerful complementary analytical tool. When integrated with other physical and forensic aspects, it can significantly assist fire investigators in ruling out external ignition hypotheses in agricultural storage incidents.^1,6^

**Figure 7:**
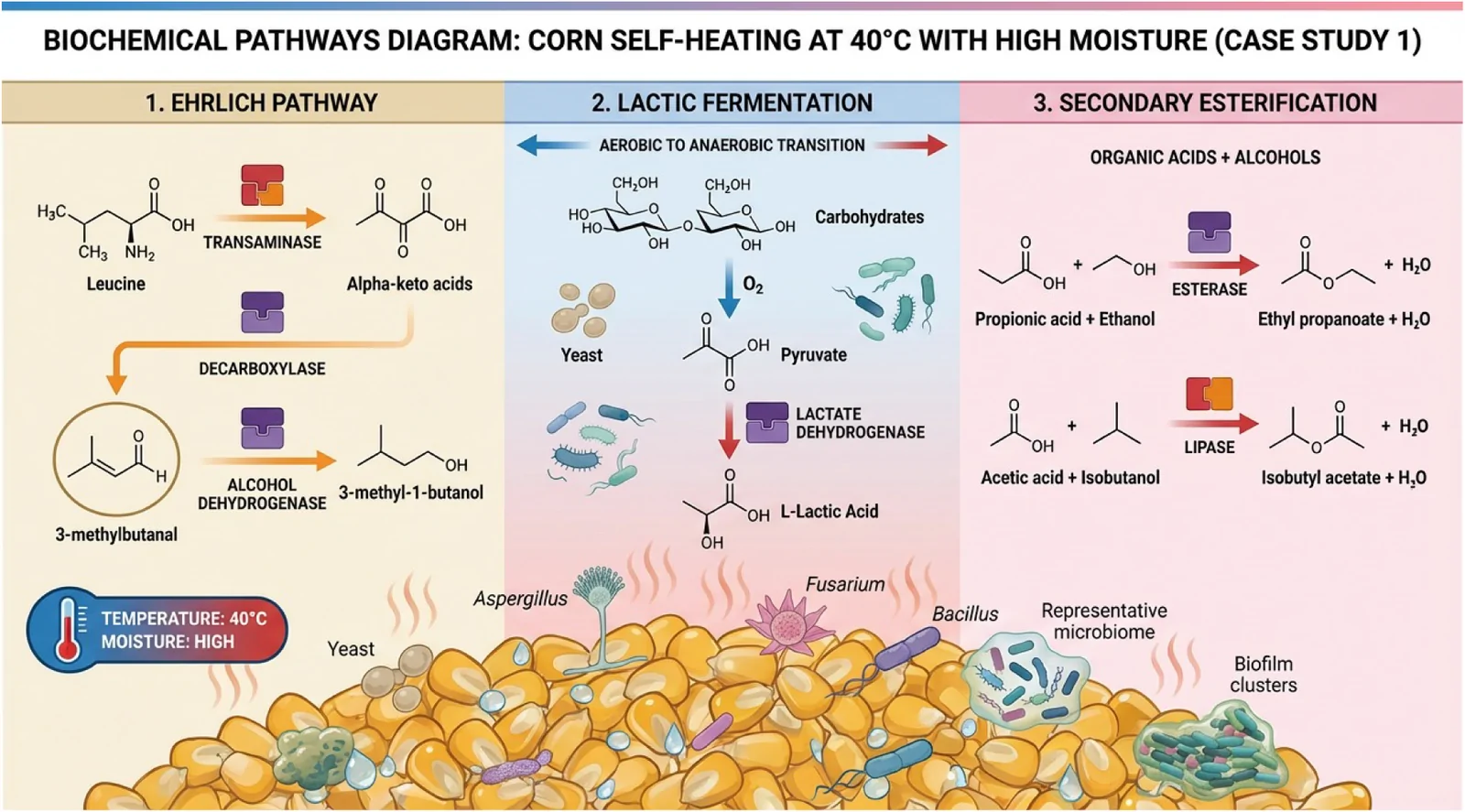
Proposed biochemical mechanisms for the initial stage of self-heating in Case Study 1 (high moisture and 40 °C). The diagram details the metabolism of the microbiota inherent to the grains, illustrating the degradation of amino acids via the Ehrlich pathway, the transition to lactic fermentation under oxygen depletion, and the subsequent formation of the detected volatile esters.

**Figure 8:**
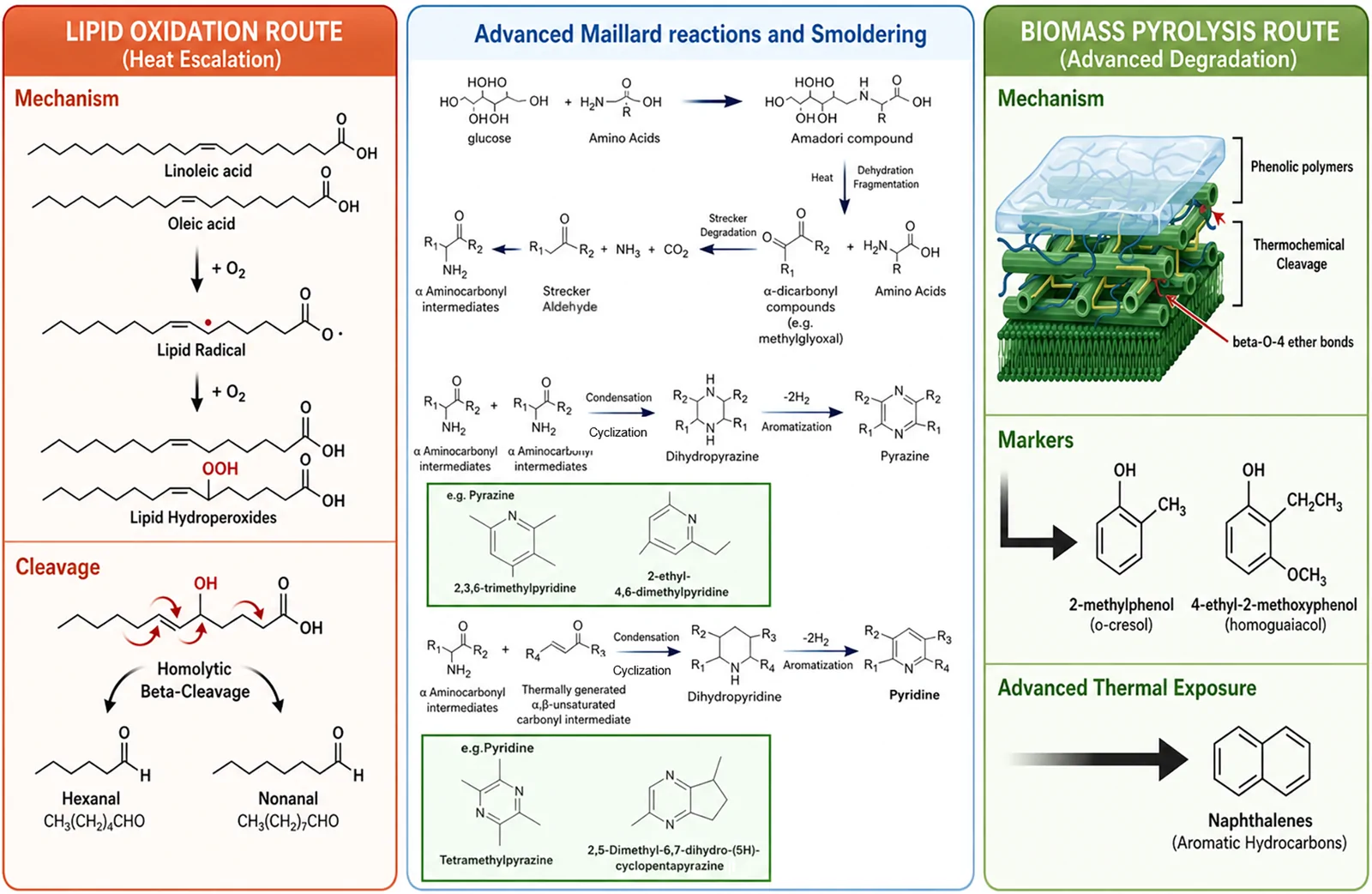
Proposed strictly thermochemical reaction mechanisms for the escalation of self-heating in Case Study 2. The pathways illustrate the transition from an abiotic thermal trigger (lipid auto-oxidation) induced by the drying temperatures to the final thermochemical cleavage of cell wall phenolic polymers and advanced Maillard reactions, immediately prior to smoldering combustion.

### Comparative Mechanistic Analysis: From Biological Trigger to Thermochemical Escalation (Case Study 1 vs Case Study 2)

While Case Study 1 and Case Study 2 were investigated under different field constraints and simulation parameters (40 °C with water addition versus 60 °C in an open system), the integration of their volatile profiles (Tables 1 and 2) provides a comprehensive mechanistic continuum of the spontaneous combustion phenomenon. It is important to note that the laboratory simulations presented herein are simplified models designed strictly to support the proposed chemical interpretations, rather than full-scale reproductions of the complex and dynamic scenarios found in real silo fires. Case Study 1 elucidates the biological trigger phase, typical of storage silos experiencing water infiltration, where the localized accumulation of moisture drives intense microbial metabolism.^45^ In this initial stage, the thermodynamic system is governed by enzymatic pathways, specifically the Ehrlich pathway and lactic fermentation, resulting in the prominent relative emission of short-chain alcohols, organic acids, and secondary esters.^17,31^ The latent heat generated by this microbial respiration is entrapped within the insulating grain mass, gradually raising the internal temperature.^45^ In contrast, the thermal stress simulation of Case Study 2 effectively represented the operational scenario of a dryer silo, where the machinery itself acts as an initial stressor. Once the temperature surpasses the microbial survival threshold (typically between 50 and 60 °C), the system transitions into the thermochemical escalation phase.^45^ At this tipping point, biological activity is inhibited, the open system configuration promotes the gradual evaporation of moisture, and the accumulated biological heat (or heat imposed externally by the dryer) provides the activation energy for abiotic reactions. The mechanism shifts entirely to the homolytic cleavage and autoxidation of lipids (yielding hexanal and nonanal)^18,40^ and advanced Maillard and Strecker reactions (yielding pyrazines).^39,44,46^ Ultimately, this thermal runaway breaks the thermodynamic stability of the biomass, leading to the extreme pyrolytic degradation and smoldering combustion observed in the partially burned samples of both cases.^8,12,28^

The material evidence of this mechanistic difference lies in the chemical signatures of the partially burned (PB) samples. In Case Study 1, even at an advanced stage, the PB sample still exhibits a strong biological influence, being dominated by primary microbial metabolites (such as 3-methyl-1-butanol and lactic acid) and secondary esters (Table 1), evidencing that the collapse occurred in an environment previously conditioned by intense microbial activity and oxygen deficiency.^17,31^ In contrast, the PB sample from Case Study 2 is completely devoid of these biological markers, being exclusively dominated by advanced thermochemical products, such as tetramethylpyrazine, substituted phenols derived from thermal degradation^39,41,44^ (Table 2). This distinct chemical separation demonstrates that the elevated temperature of the drying process acted as a direct abiotic trigger, suppressing microbial metabolism and driving the matrix directly into thermo-oxidative degradation.^45^ Consequently, the results demonstrate that, although both cases result in spontaneous combustion, the mechanistic pathways are distinct: one governed by a biological trigger followed by thermal escalation (Case 1), and another initiated directly by thermochemical conditions (Case 2), as synthesized in Figures 7 and 8. This distinction is critically relevant from a forensic standpoint, as it evidence different root causes, preserving unique chemical signatures even after burning and partial carbonization.^8,12^

## Conclusions

Distinct profiles of volatile organic compounds (VOCs) were identified, allowing the proposal of a chronological mapping of the self-heating and *smoldering* combustion processes in stored corn. The detection of alcohols, organic acids, and nitrogenous heterocyclic compounds provided supportive chemical evidence of intense microbial activity, indicating the transition to anaerobic deterioration conditions that precede ignition. In subsequent stages, the conversion of esters associated with the formation of aldehydes, phenols, nitrogenous heterocycles, and aromatic hydrocarbons reflects the thermal escalation resulting from lipid oxidation reactions and the progressive thermochemical degradation (pyrolysis) of the biomass matrix. The comparative analysis by HS-GC-MS demonstrated its potential as a valuable tool for forensic investigation by allowing a reconstruction of the chemical evolution of the event based on the chemical markers generated in the different degradation stages.

Furthermore, the comparative evaluation between the two case studies provided valuable insights into the distinct mechanisms driving the self-heating progression. While the 40 °C scenario (Case Study 1) highlighted the biological trigger through the emission of primary alcohols and organic acids derived from microbial metabolism, the 60 °C scenario (Case Study 2) demonstrated how temperatures lethal to the microbiota shift the process toward strictly abiotic thermochemical reactions, primarily dominated by lipid auto-oxidation and subsequent structural degradation.

The comparative analysis by HS-GC-MS proved to be a valuable tool for forensic investigation by allowing the reconstruction of the chemical evolution of the event based on the chemical markers generated in the different degradation stages. This approach contributes to the understanding of self-heating mechanisms in stored biomass and, when integrated with the systematic investigation method proposed by NFPA 921, strengthens the scientific reliability in determining the causes of fires in agricultural storage systems.^1^

As future perspectives, in order to overcome the inherent limitations of library-based identification in complex agricultural matrices, subsequent studies should focus on the quantitative validation of these key biomarkers using authentic analytical standards, calculation of retention indices (RI), or the application of advanced multidimensional chromatography and detailed analysis of the mass spectra of each identified peak.

Furthermore, to fully elucidate the biological triggers of the self-heating process, the fermentation simulations should be complemented by the molecular identification of the deteriorating microbiota. Conducting isolated *in vitro* fermentation assays using specific yeast strains (such as *Saccharomyces cerevisiae*) is recommended to quantitatively monitor the exact emission pathways of these primary volatile precursors in corn grains.

## Supplementary Material



## Notes

Experimental conditions for HS-GC-MS analysis of samples collected in Case Study 1 (Table S1); experimental conditions for HS-GC-MS analysis of samples collected in Case Study 2 (Table S2); schematic representation of the headspace gas chromatography (HS-GC) methodology, illustrating sample incubation in a sealed vial, partitioning of volatile compounds into the headspace, transfer via a six-port valve and sampling loop, and injection into the gas chromatograph (Figure S1); HS-GC-MS chromatograms of corn samples for Case Study 1: (a) blank sample (without corn), (b) control sample, (c) visually intact corn grains sampled from a distant area within the same silo (conveyor belt), containing approximately 5% charred grains after fermentation simulation, (d) replicate of visually intact corn grains sampled from a distant area within the same silo (conveyor belt), containing approximately 5% charred grains after fermentation simulation, (e) partially burned sample, and (f) burned sample (Figure S2); relative abundance (area %) of volatile organic compounds identified by HS-GC-MS in corn samples from Case Study 1 after fermentation simulation and partial burning, with intact corn after fermentation simulation and partially burned corn, replicate samples omitted for clarity, and only compounds detected in both samples shown to facilitate comparison (Figure S3); relative abundance (area %) of volatile organic compounds identified by HS-GC-MS in corn samples from Case Study 2: (a) moist corn, (b) partially burned corn, (c) moist corn after 3 days of fermentation, and (d) moist corn after 7 days of fermentation (Figure S4); relative abundance (area %) of volatile organic compounds identified by HS-GC-MS in corn samples from Case Study 2, including moist corn, partially burned corn, and moist corn after 3 and 7 days of fermentation (Figure S5); visual comparison of moist corn samples (a) before aging and after aging for (b) 3 days and (c) 7 days under controlled conditions (Figure S6) (PDF)
