# Supplementary material for: Chemical Indicators of Self-Heating and Spontaneous Combustion in Stored Grains Investigated by HS-GC-MS
Source: ACS Omega. 2026 Aug 7;11(32):47804–16. doi: 10.1021/acsomega.6c03108 (PMC13491511; doi:10.1021/acsomega.6c03108)
Supplement: Supplementary file 1 [file ao6c03108_si_001.pdf]

Supporting Information for *Chemical Indicators of Self-Heating and Spontaneous Combustion in Stored Grains Investigated by HS-GC-MS*

Paula Baptista Leme, Gustavo Cabral Ferraz, Teresa Cristina Brazil Paiva, Maurício Lamano Ferreira, Eliane Corrêa Pedrozo*, Flávio Teixeira da Silva**

School of Engineering of Lorena, University of São Paulo (USP), Lorena, São Paulo, Brazil.

Table S1. Experimental conditions for HS-GC-MS analysis of samples collected in case study 1.

Chromatografic Column: DB-5MS- UI - 30 m × 0.25 mm i.d., coating 0.25 μm		
CG oven tempetature program		
Heating rate (˚C.min-1)	Temperature (˚C)	Isotermal hold (min)
-	40	5
11	300	10
Injector temperature: 250 ˚C		Incubation temperature: 85 ˚C
Interface temperature: 280˚C		Incubation time: 60 min
Detector temperature: 280 ˚C		Injection volume: 1000 μL
Carrier gas: Helium		Split ratio = 1:5

Table S2. Experimental conditions for HS-GC-MS analysis of samples collected in case study 2.

Chromatografic Column: DB-5MS- UI - 30 m × 0.25 mm i.d., coating 0.25 μm		
CG oven tempetature program		
Heating rate (˚C.min-1)	Temperature (˚C)	Isotermal hold (min)
-	50	5
11	300	10
Injector temperature: 250 ˚C		Incubation temperature: 85 ˚C
Interface temperature: 280˚C		Incubation time: 60 min
Detector temperature: 280 ˚C		Injection volume: 1000 μL
Carrier gas: Helium		Split ratio = 1:5

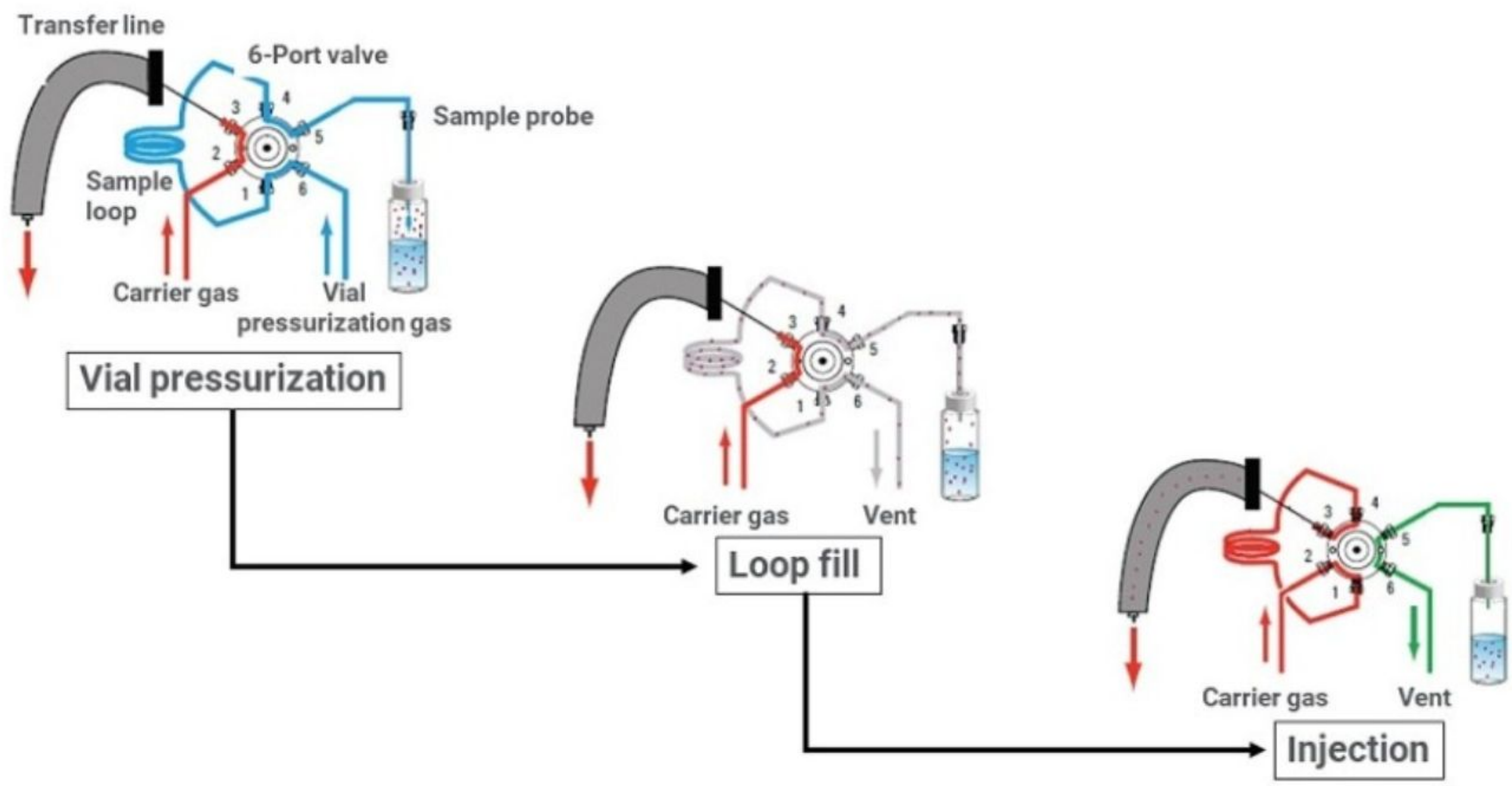

Figure S1. Schematic representation of the headspace gas chromatography (HS-GC) methodology, illustrating sample incubation in a sealed vial, partitioning of volatile compounds into the headspace, transfer through a 6-port valve and sample loop, and injection into the gas chromatograph.

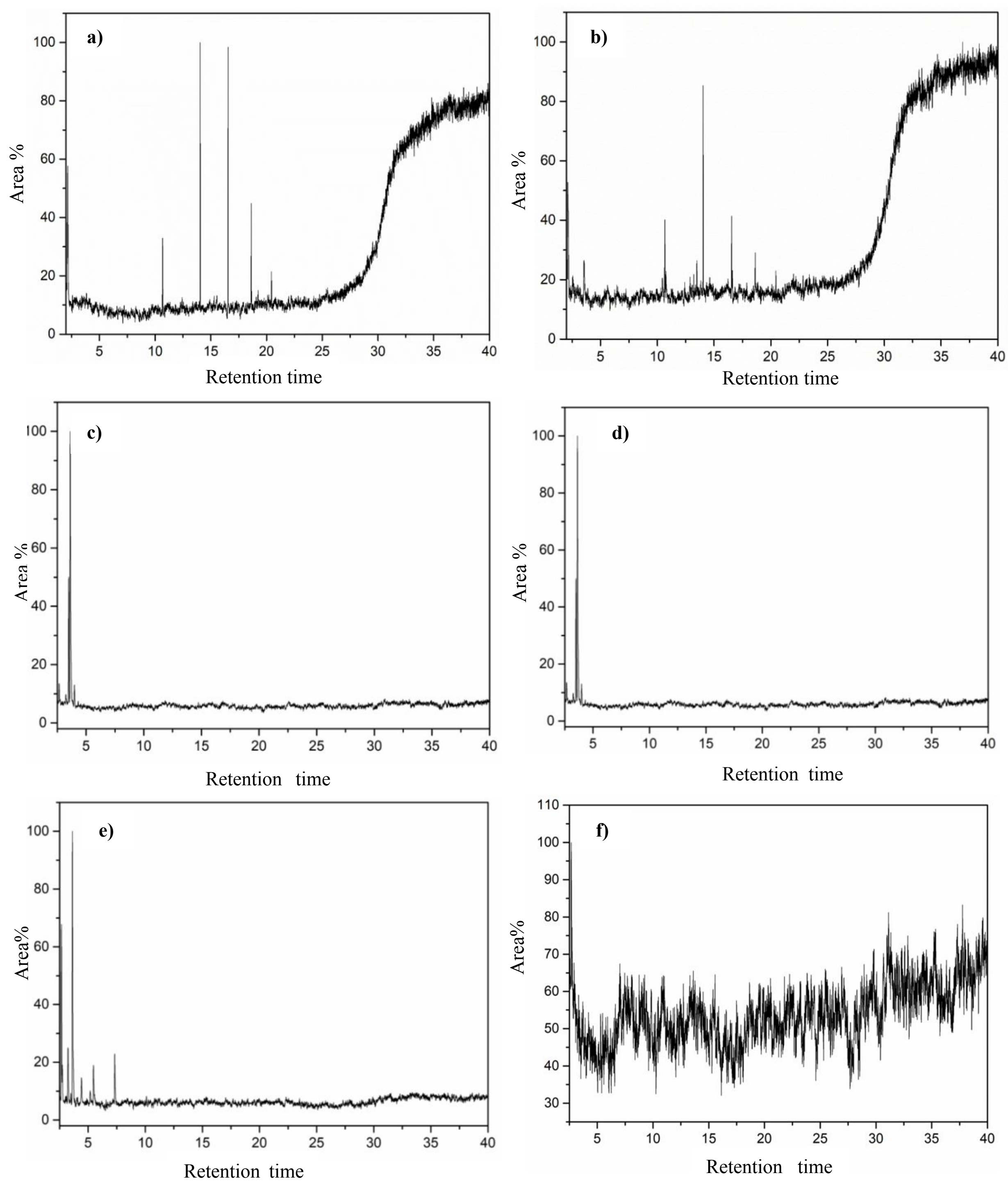

Figure S2. HS-GC-MS chromatograms of corn samples for Case Study 1: (a) blank sample (without corn), (b) control sample, (c) visually intact corn grains sampled from a distant area within the same silo (conveyor belt), containing approximately 5% charred grains after fermentation simulation, (d) replicate of visually intact corn grains sampled from a distant area within the same

silo (conveyor belt), containing approximately 5% charred grains after fermentation simulation, (e) partially burned sample, and (f) burned sample.

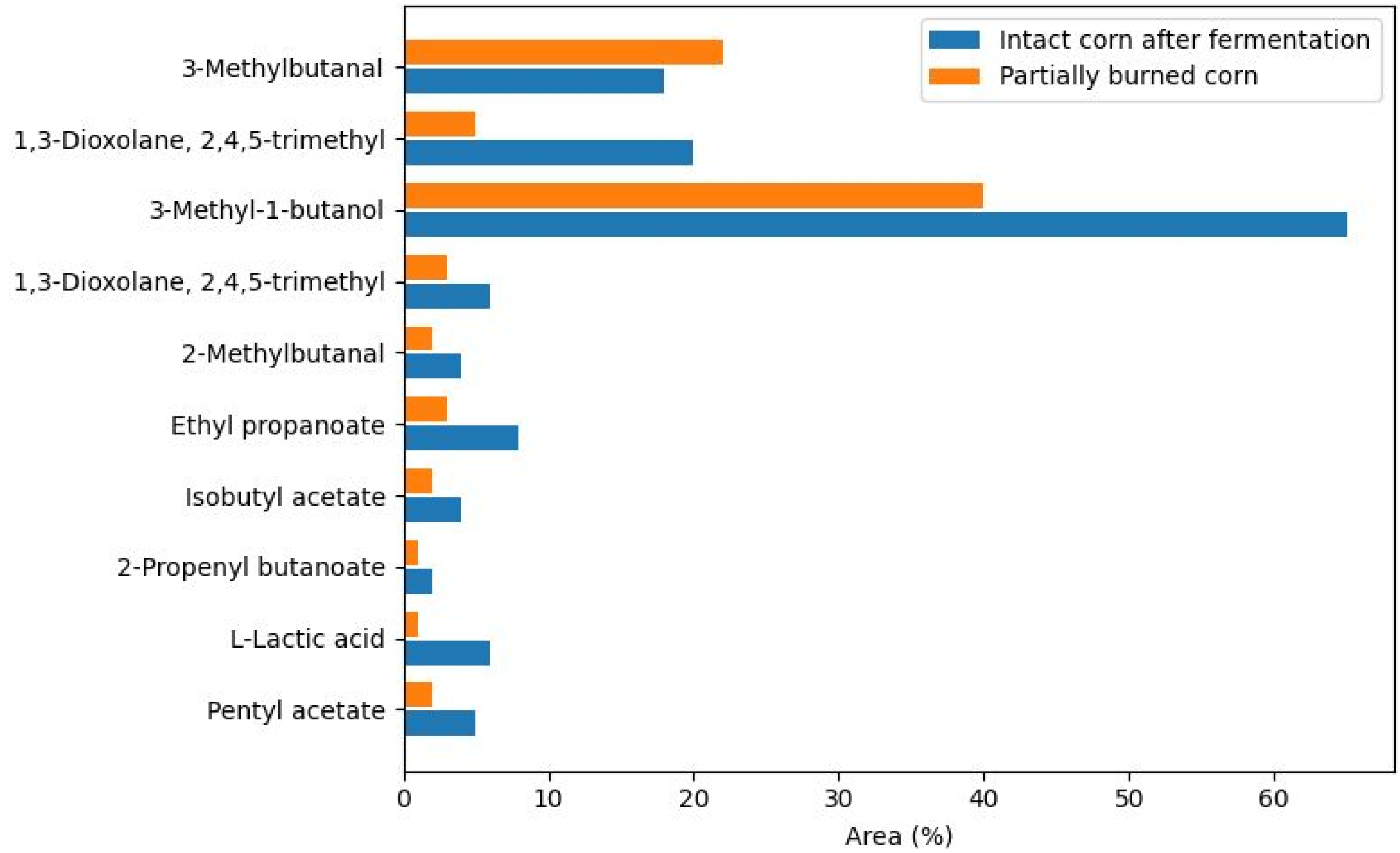

Figure S3. Relative abundance (area %) of volatile organic compounds identified by HS-GC-MS in corn samples from Case Study 1 after fermentation simulation and partial burning. The figure presents intact corn after fermentation simulation and partially burned corn. Replicate samples were omitted for clarity, and only compounds detected in both samples are shown to facilitate comparison.

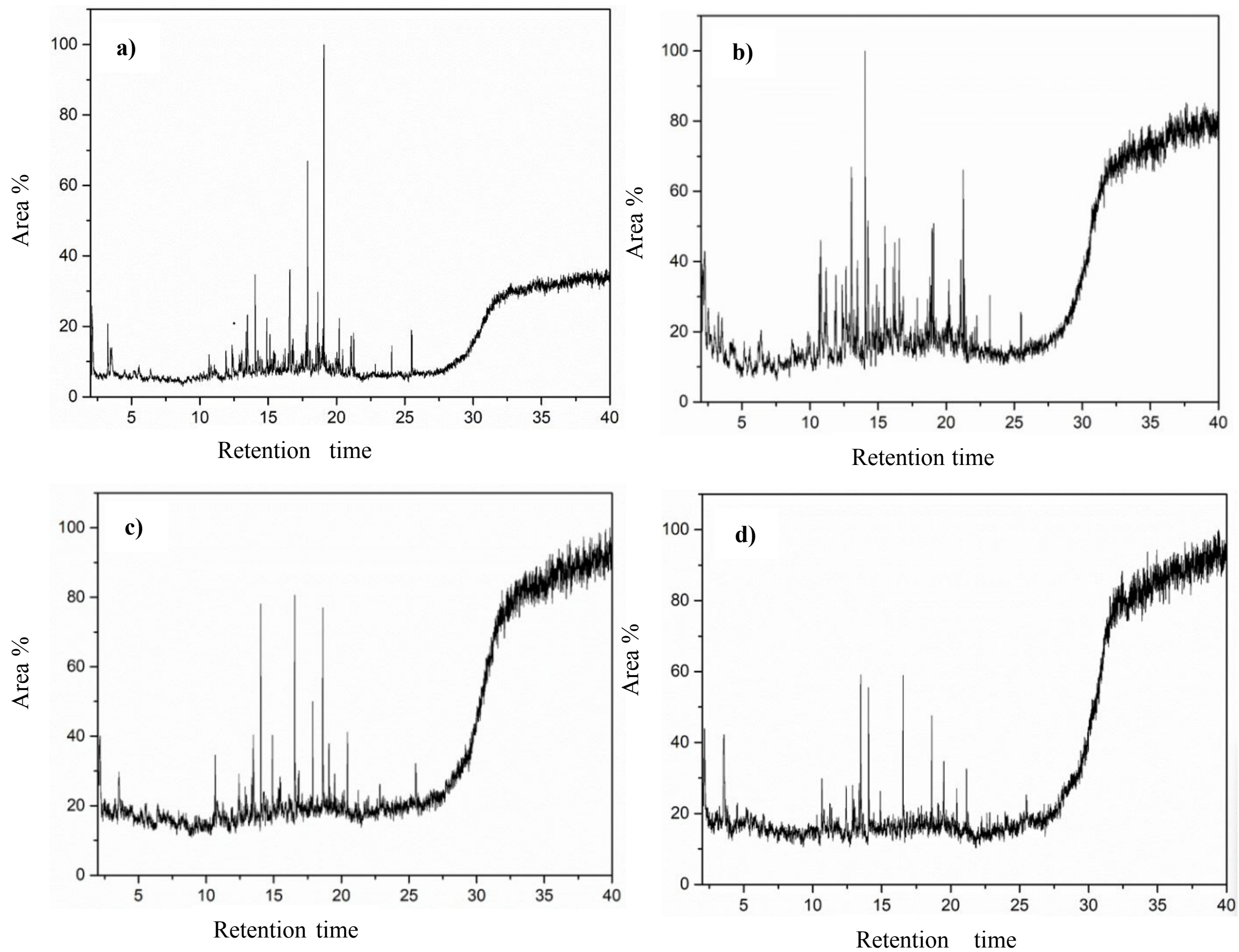

Figure S4. Relative abundance (area %) of volatile organic compounds identified by HS-GC-MS in corn samples for case study 2 a) moist corn b) partially burned corn c) moist corn after 3 days of fermentation d) moist corn after 7 days of fermentation.

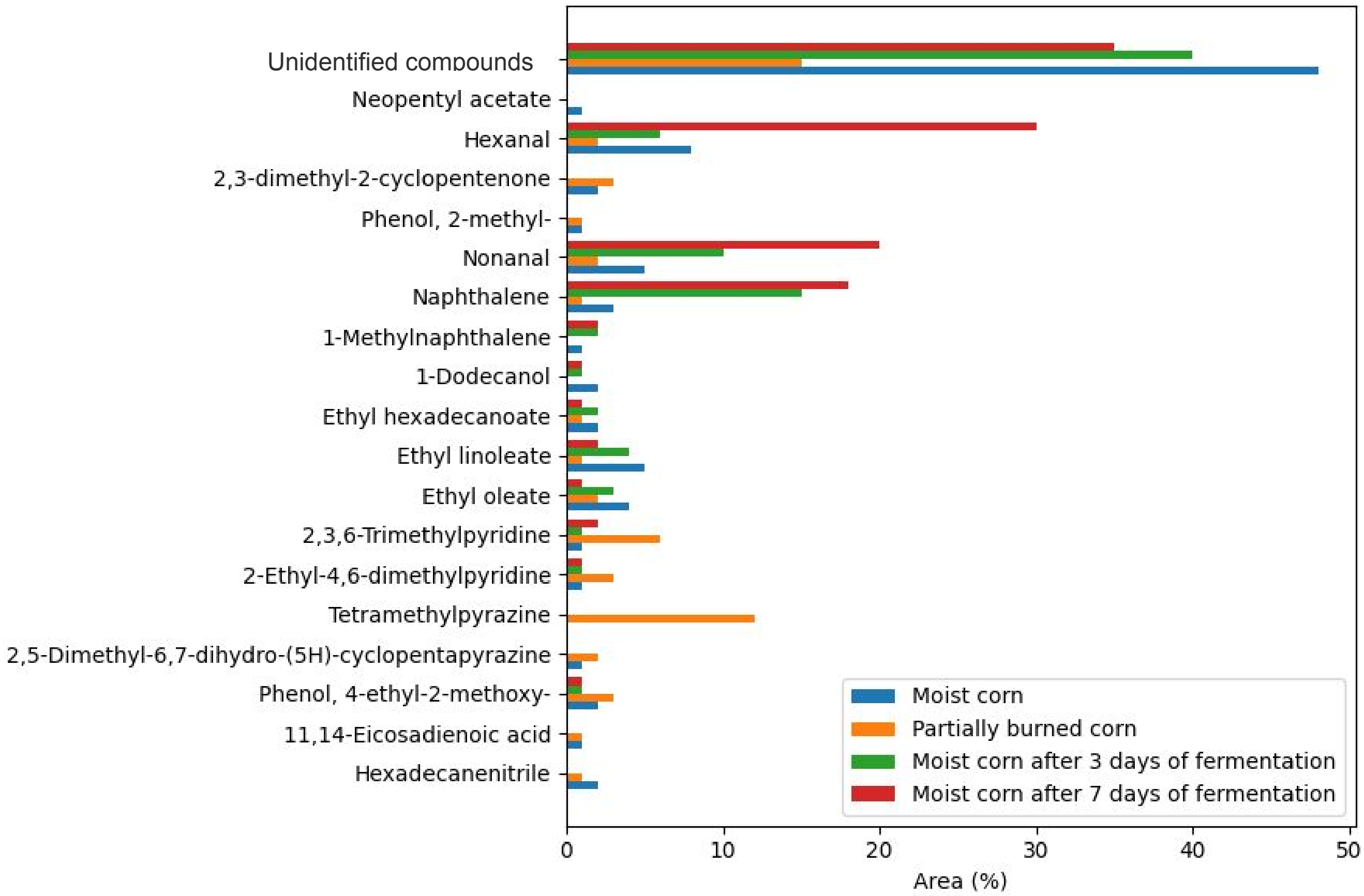

Figure S5. Relative abundance (area %) of volatile organic compounds identified by HS-GC-MS in corn samples from case study 2, including moist corn, partially burned corn, and moist corn after 3 and 7 days of fermentation.

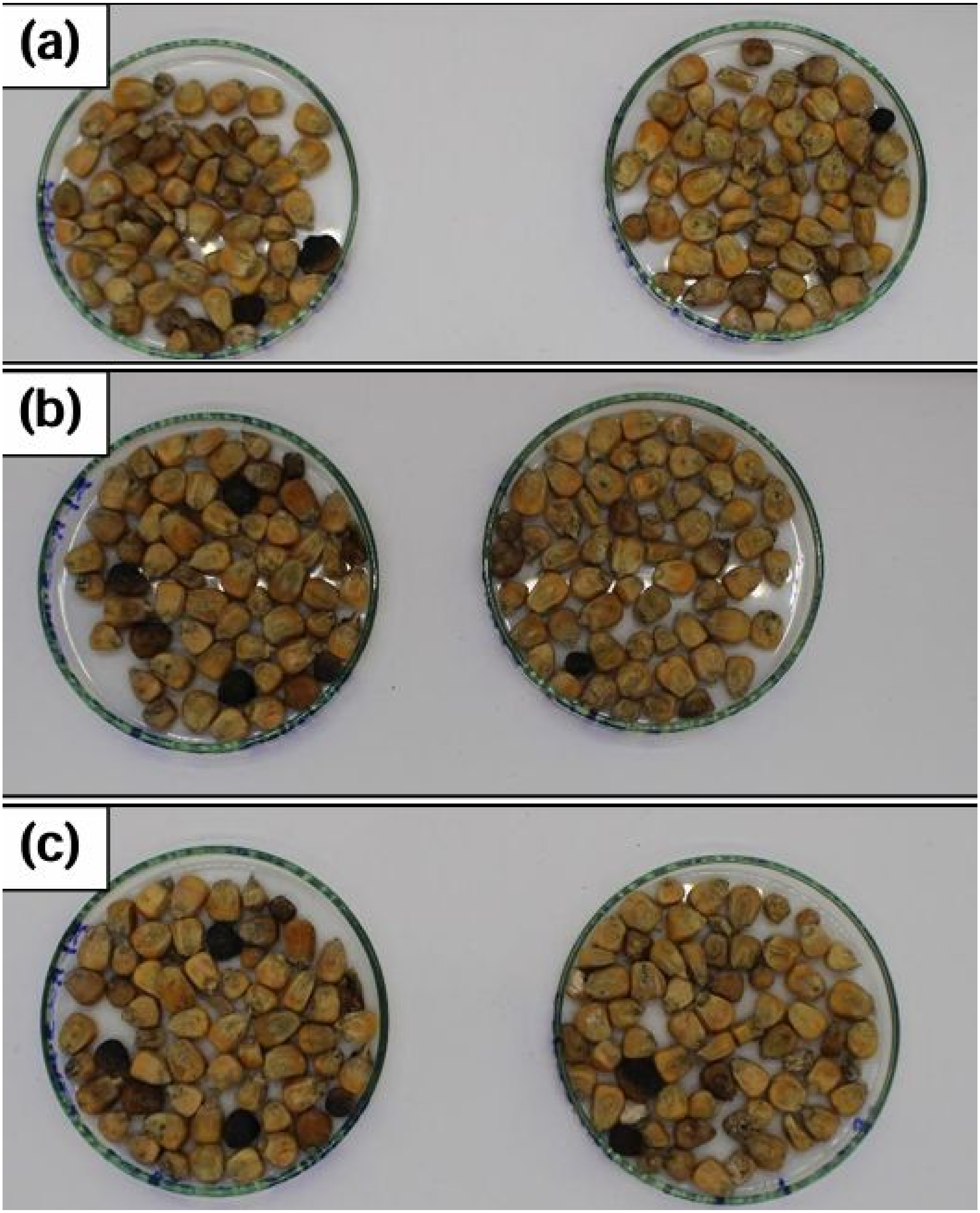

Figure S6. Visual comparison of moist corn samples (a) before aging and after aging for (b) 3 days and (c) 7 days under controlled conditions.
